# Diabetic Myonecrosis: A Diagnostic and Treatment Challenge in Longstanding Diabetes

**DOI:** 10.1155/2018/1723695

**Published:** 2018-08-12

**Authors:** Lima Lawrence, Oscar Tovar-Camargo, M. Cecilia Lansang, Vinni Makin

**Affiliations:** ^1^Cleveland Clinic, Department of Endocrinology and Metabolism, Cleveland, OH, USA; ^2^Cleveland Clinic, Department of Anesthesiology, Cleveland, OH, USA

## Abstract

**Objective:**

Diabetes mellitus is associated with microvascular and macrovascular complications; the most commonly recognized ones include diabetic nephropathy, retinopathy, and neuropathy. Less well-known complications are equally important, as timely recognition and treatment are essential to decrease short- and long-term morbidity.

**Methods:**

Herein, we describe a case of a 41-year-old female with longstanding, uncontrolled type 2 diabetes, who presented with classical findings of diabetic myonecrosis.

**Results:**

Our patient underwent extensive laboratory and imaging studies prior to diagnosis due to its rarity and similarity in presentation with other commonly noted musculoskeletal conditions. We emphasize the clinical presentation, laboratory and imaging findings, treatment regimen, and prognosis associated with diabetic myonecrosis.

**Conclusion:**

Diabetic myonecrosis is a rare complication of longstanding, poorly controlled diabetes mellitus. The diagnosis requires a high index of suspicion in the right clinical setting: acute onset nontraumatic muscular pain with associated findings on clinical exam, laboratory studies, and imaging. While the short-term prognosis is good, the recurrence rate remains high and long-term prognosis is poor given underlying uncontrolled diabetes and associated sequelae.

## 1. Introduction

Diabetic myonecrosis, also known as diabetic muscle infarction, causes spontaneous ischemic necrosis of skeletal muscle most commonly in the thigh or calf. It is a rare complication seen in patients with longstanding, uncontrolled diabetes mellitus. It was first described in 1965 by Angervall and Sterner as “tumoriform focal muscular degeneration” [1]. Patients present with acute pain, swelling, and tenderness of the affected muscle group [2]. As diabetic myonecrosis is infrequently seen, heightened awareness of the condition is necessary to exclude similarly presenting muscular and vascular pathology and promptly initiate treatment.

## 2. Clinical Case

A 41-year-old African American woman presented to the emergency department (ED) with right leg pain for 2 weeks. She had a past medical history of type 2 diabetes mellitus diagnosed more than 10 years ago, end-stage renal disease (ESRD) on hemodialysis, hypertension, congestive heart failure, and recently resolved left lower extremity cellulitis. She described her right leg pain as constant, aching, stabbing pain in the right posterior mid-thigh with radiation distally to the calf. She denied any trauma or falls and reported worsening pain with weight-bearing and ambulation. She had already presented to 2 other EDs and had X-ray of the right knee and lumbar spine, venous Doppler of the right lower extremity, CT femur and right ankle-brachial index, which were normal. She had been taking oxycodone-acetaminophen without significant relief. During the current visit, she had CT angiogram of the abdomen and pelvis with lower extremity runoff, which found no vessel stenosis, but noted soft tissue and fascial edema in the right thigh. She was discharged home with analgesics and recommended follow-up with orthopedics.

The following month, patient presented to the ED again with excruciating right thigh pain. Laboratory studies were remarkable for leukocytosis 12.77 k/uL (3.7–11.0 k/uL), elevated creatinine kinase (CK) 683 U/L (42–196 U/L), C-reactive protein (CRP) 3.7 mg/dL (<0.9 mg/dL), and erythrocyte sedimentation rate (ESR) 68 mm/hr (0–20 mm/hr). Additionally, poor glycemic control was confirmed with random blood glucose of 569 mg/dL and hemoglobin A1c 13.8%. MRI of the right leg revealed diffuse subcutaneous edema in the right thigh, extending to the level of the knee, with diffusely increased T2 signal in the mid and distal thigh. Intramuscular fascial edema around the proximal hamstring muscles was noted, without any findings of abscess or osteomyelitis. Patient received analgesics, with optimization of glycemia, and was discharged home after physical therapy evaluation.

**Figure 1:**
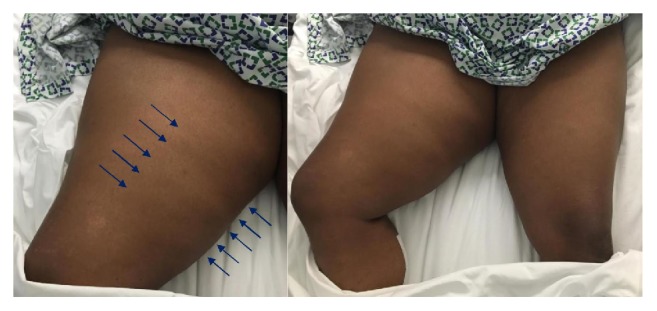
Diabetic myonecrosis involving the right thigh with overlying palpable skin induration (arrows). Enlargement of right thigh compared to left. Right leg was kept externally rotated.

**Figure 2:**
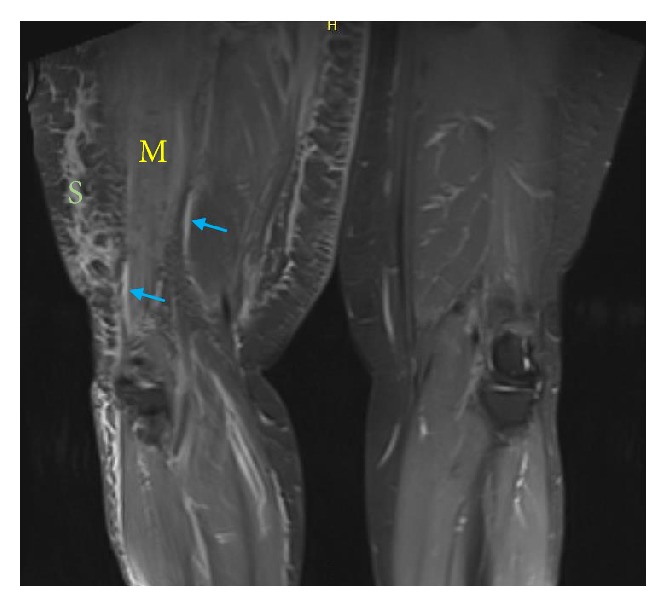
**MRI leg without contrast.** Right thigh musculature (M) and subcutaneous tissues (S) with diffuse swelling and edema-like signal on T1-weighted imaging. Fluid-like signal is noted at the fascial planes (arrows).

One week later, patient was readmitted after a fall. Endocrinology was consulted to address hyperglycemia. Physical examination revealed an obese woman in mild distress due to pain. She had a swollen right thigh, exquisitely tender to palpation and noticeably larger than the left. The overlying skin was palpably indurated without warmth, erythema, bullae, greyish hue, or crepitus (Figure 1). Passive and active movements at the right hip and knee were limited due to pain and patient kept the right leg externally rotated. No visible cord or joint effusions at the knee or hip were noted and lower extremity pulses were palpable bilaterally. Laboratory studies were notable for persistent leukocytosis 13.60 k/uL, elevated CRP 8.4 mg/dL, ESR 117 mm/hr, and CK 714 U/L. As the inflammatory markers doubled in a short interval, repeat lower extremity MRI was obtained to rule out abscess or infectious myositis. T1-weighted imaging on MRI noted diffuse swelling and edema-like signal involving the right thigh musculature with fluid-like signal at the fascial planes without any focal fluid collection (Figure 2). Altogether, these findings were suggestive of ischemic changes in the right thigh musculature. Based on the clinical history as well as labs and imaging findings, a diagnosis of diabetic myonecrosis was made. Patient was prescribed aspirin 81 mg daily, analgesics including acetaminophen and oxycodone as needed (with avoidance of NSAIDs due to ESRD), and lidocaine patch. Patient's blood glucose was targeted from 140 to 180 mg/dL with adjustments of insulin glargine and lispro. She was evaluated by physical therapy and discharged home shortly with endocrine follow-up.

## 3. Discussion

Diabetic myonecrosis is infrequently observed in patients with diabetes. The pathogenesis of diabetic muscle infarction is poorly understood, but various theories have been proposed including vasculopathic changes from longstanding, poorly controlled diabetes, vasculitic changes, hypercoagulability, or ischemia-reperfusion injury. Microvascular endothelial damage leads to tissue ischemia, triggering the inflammatory cascade leading to ischemic necrosis. Reperfusion of necrotic tissues leads to generation of reactive oxygen species and production of inflammatory mediators including tumor necrosis factor and platelet-activating factor, which mediate vasculopathic changes [2]. Additionally, alterations in the coagulation-fibrinolysis system have been implicated in diabetic myonecrosis by causing hypercoagulability and vascular endothelial damage [2].

Diabetic myonecrosis should be suspected in any patient with diabetes who presents with sudden-onset muscle swelling and pain, particularly of the proximal lower extremities. A higher index of suspicion should be reserved for poorly controlled, longstanding diabetes patients with coexisting complications. Diabetic myonecrosis is more commonly seen in women (53.7–61.5%) with a mean age at presentation around 42.6 to 44.5 years [2–4]. A systematic review examined 126 cases of diabetic myonecrosis and observed that the mean diabetes duration at the time of diagnosis was 18.9 years in type 1 diabetes and 11.0 years in type 2 diabetes. Additionally, mean hemoglobin A1c at the time of diagnosis was 9.34%. Similarly, an examination of diabetic myonecrosis in 41 patients with ESRD found that more than 60% of patients had hemoglobin A1c above 7.0% [3]. Coexisting diabetes complications are frequently noted, with nephropathy in 75% of patients, two other macrovascular complications in 65.8% of patients, and 46.6% with nephropathy, neuropathy, and retinopathy related to diabetes [2].

Initial symptoms most commonly present in the thigh and calf, followed by upper extremity sites. The most commonly affected muscle groups are found in the anterior thigh, followed by the calf and posterior thigh [2]. Painful swelling may be acute or evolve over days to weeks. There is usually no preceding trauma and bilateral involvement is uncommon [4]. Although there are no specific markers, a common pattern is noted with elevation of CK, ESR, and CRP. Leukocytosis may be noted, although most patients are afebrile on presentation. Diagnosis is based on clinical presentation, labs, and imaging, for which MRI is the modality of choice. Characteristic features on MRI include increased signal intensity in the affected intramuscular and subcutaneous tissues, hyperintense signal on T2-weighted imaging, and isointense to hypointense signal on T1-weighted images associated with inflammatory changes and edema [5]. Doppler ultrasound is commonly performed to exclude underlying deep venous thrombus (DVT) or abscess and may visualize subcutaneous or muscle edema. Additional imaging is not necessary but may be performed to exclude other diagnoses as appropriate. Differential diagnoses include DVT, pyomyositis, necrotizing fasciitis, soft tissue abscess, ruptured Baker's cyst, osteomyelitis, and benign tumors or muscle sarcomas [6]. Muscle biopsy must be reserved for atypical presentation, uncertain diagnosis, or when treatment fails to improve symptoms. On biopsy, gross findings include nonhemorrhagic and pale muscle tissue. Under light microscopy, initial stages of diabetic myonecrosis reveal areas of muscle necrosis and edema, with replacement of necrotic muscle fibers by fibrous tissue, lymphocytic infiltration, and muscle regeneration in later stages [3, 5].

Rest, analgesia, and intense glycemic control are the cornerstones of diabetic myonecrosis therapy. As no randomized trials are available comparing specific treatment regimens, recommendations are based on case reports and case series. Initially, bed rest is advised as patients receiving physical therapy were noted to have the longest mean time to symptom resolution [2]. Low-dose aspirin and NSAIDs are recommended for those without contraindication; however, it is important to note that a large majority have concomitant renal disease, necessitating evaluation of NSAID therapy on a case-by-case basis. The benefits of antiplatelet and anti-inflammatory agents in diabetic myonecrosis may be attributed to its antithrombotic effects and amelioration of endothelial dysfunction. Surgical intervention is not routinely recommended as recovery time and recurrence rate were highest in this group [2]. With timely diagnosis and initiation of treatment, diabetic myonecrosis resolves spontaneously over a few weeks to months. Average recovery times were 5.5 weeks with aspirin and/or NSAID use, 8 weeks with bed rest and analgesics, and 13 weeks with surgical resection [7]. Even with treatment, diabetic myonecrosis carries a high recurrence rate of 34.9 to 47.8% usually involving a contralateral limb within 6 months [2, 3, 5]. Additionally, the long-term outlook remains poor with high morbidity and mortality, given underlying diabetes complicated by severe end-organ disease. A 25-year Mayo Clinic experience review revealed that out of 5 patients with diabetic myonecrosis, one required kidney transplantation, and a second patient died 2 years after diagnosis of myonecrosis [8].

## 4. Conclusion

Diabetic myonecrosis is a rare complication of longstanding, poorly controlled diabetes mellitus. Diagnosis requires a high index of suspicion in the right clinical setting: acute onset nontraumatic muscular pain with associated findings on clinical exam, labs, and imaging. Muscle biopsy is not routinely indicated and treatment is mainly conservative with good short-term prognosis and resolution of symptoms. However, the recurrence rate remains high, and long-term prognosis is poor given underlying uncontrolled diabetes and its associated sequelae.

## References

[1] Angervall L., Stener B. (1965). Tumoriform focal muscular degeneration in two diabetic patients. *Diabetologia*.

[2] Horton W., Taylor J., Ragland T. (2015). Diabetic muscle infarction: a systematic review. *BMJ Open Diabetes Research & Care*.

[3] Yong T., Khow K. (2018). Diabetic muscle infarction in end-stage renal disease: A scoping review on epidemiology, diagnosis and treatment. *World Journal of Nephrology*.

[4] Joshi T., D'Almeida E., Luu J. (2015). Diabetes myonecrosis - A rare complication. *Diabetes Research and Clinical Practice*.

[5] Trujillo-Santos A. J. (2003). Diabetic muscle infarction: an underdiagnosed complication of long-standing diabetes. *Diabetes Care*.

[6] Chawla A., Dubey N., Chew K. M., Singh D., Gaikwad V., Peh W. C. (2017). Magnetic resonance imaging of painful swollen legs in the emergency department: a pictorial essay. *Emergency Radiology*.

[7] Kapur S., Brunet J., McKendry R. (2004). Diabetic muscle infarction: case report and review. *The Journal of Rheumatology*.

[8] Bunch T. J., Birskovich L. M., Eiken P. W. (2002). Diabetic myonecrosis in a previously healthy woman and review of a 25-year Mayo clinic experience. *Endocrine Practice*.

